# Acupuncture for common cold: A systematic review and meta-analyze protocol

**DOI:** 10.1097/MD.0000000000010061

**Published:** 2018-03-09

**Authors:** Ying Cheng, Bifeng Gao, Yuhao Jin, Na Xu, Taipin Guo

**Affiliations:** a Yunnan University of Traditional Chinese Medicine, Kunming, Yunnan Province; b Affiliated Hospital of Shaanxi University of Traditional Chinese Medicine, Xianyang, Shaanxi, China.

**Keywords:** acupuncture, common cold, protocol, systematic review

## Abstract

**Background:**

The common cold (CC) is the most common syndromes of infection in human beings, but there is currently no special treatment. For this reason, acupuncture is used to relieve the symptoms of the CC. Acupuncture is a traditional Chinese medicine (TCM) therapy that has been used for over 2000 years to treat various diseases. However, few studies have provided evidence for the efficacy and safety of acupuncture for the CC. This study aims to evaluate the effectiveness and safety of acupuncture on CC periods and its symptoms.

**Methods:**

The following electronic databases will be searched for studies conducted through January 1, 2019: Web of Science, Cochrane Library, EBASE, World Health Organization International Clinical Trials Registry Platform, Springer, Wan-fang database, Chinese Biomedical Literature Database (CBM), Chinese Scientific Journal Database (VIP), China National Knowledge Infrastructure (CNKI), and other sources. All randomized controlled trials on acupuncture for common cold will be included. Risk of bias will be assessed using the Cochrane risk of bias assessment tool, while RevMan V.5.3.5 software will be implemented for the assessment of bias risk, data synthesis, subgroup analysis, and meta-analyses if conditions are met. Continuous outcomes will be presented as mean difference (MD) or standard mean difference (SMD), while dichotomous data will be expressed as relative risk.

**Results:**

A high-quality synthesis of current evidence of acupuncture for CC will be stated from several aspect using subjective reports and objective measures of performance. The reduction rate of common cold symptoms after initial treatment, resolved cold symptoms, and reduced cold duration will be collected.

**Conclusion:**

This protocol will present the evidence of whether acupuncture therapy is an effective intervention for CC.

## 1 Introduction

Respiratory viral infections, also known as the common cold (CC), are the ubiquitous syndrome of infection in human beings.^[1]^ However, it is also the main cause of morbidity and mortality on a worldwide basis.^[2]^ It is a spontaneously remitting infection of the upper respiratory tract, characterized by a runny nose, nasal obstruction, headache, sneezing, cough, and fever (usually <37.8 °C).^[3]^ There is uncertainty regarding the efficacy and safety of interventions for preventing and treating the common cold in healthy people because the frequent alterations in the antigenic structures of respiratory viruses. Further, even bacteria act as infective agents.

Accordingly to the research,^[4]^ from 30% to 50% of cases of cold syndrome are caused by rhinoviruses, but the etiology can vary depending on the epidemiological situation. Bacterial infections were rare, suggesting that the common cold is almost exclusively a viral infection. So far our understanding of the mechanisms that generate cold symptoms is limited. Today, conventional methods of medications, such as analgesic agents and antihistamines are only effective for the alleviation of symptoms, and antibiotic treatment is not justified.^[5]^

Acupuncture is prevalent in South Asia like China, Korea, and Japan. The main theories are based on Yin-Yang, Qi, meridian and acupoints. Each acupoint has its own specific therapeutic functions, and the prescription of acupoints is formulated by different acupoints and special stimuli ways based on ancient Chinese Medicine theory.^[6]^

Since the early 1970s, acupuncture began to spread in West, and almost 500 randomized controlled trials (RCTs) have evaluated its efficacy.^[7]^ In 1997, a National Institutes of Health Consensus Development Panel on acupuncture reached the conclusion^[8]^ that there is clear evidence that needle acupuncture is efficacious for many diseases, particularly in relief of pain on diverse pain conditions.

The effect mechanism of common cold by acupuncture has not been fully found so far. However, accumulated evidences have shown acupuncture may inhibit early-phase vascular permeability,^[9]^ impair leukocyte adherence to vascular endothelium, and suppress exudative reaction a degree equivalent to that of orally administered aspirin and indomethacin.^[10]^ Evidence also supports that there was an adjusting-network of immune in the body, and acupuncture therapy can heighten the cellular immune function of patients and provide a beneficial effect by affecting the levels of T lymphocytes subgroup (CD3+, CD4+, CD8+), soluble interleukin-2 receptor (SIL-2R), and beta-endorphin (beta-EP) in the peripheral blood of patients.^[11]^ It was also reported that acupuncture has detoxification effect through the amelioration of antioxidant defense systems.^[12]^

The research will summarize the acupuncture therapy methods as intervention for common cold, to evaluate the therapeutic value of acupuncture and provide clinical decision scheme for clinicians.

## 2 Methods

This systematic review protocol has been registered in the PROSPERO international prospective register of systematic reviews (CRD 42018085862).

### 2.1 Selection criteria

#### 2.1.1 Types of studies

RCT and blinded related research will be included only. All original studies in English or Chinese that reported the effectiveness and/or safety on acupuncture for CC will be included. We will examine RCTs that involve at least 1 test treatment that aimed to improve or eliminate CC symptoms, and 1 control treatment (or no treatment) with concurrent enrolment.

#### 2.1.2 Types of patients

Study participants diagnosed with CC must conform to diagnosis standards established by bodies such as the American Academy of Physician Pediatrics Committee or American Academy of Physician Assistant. Many other diagnostic distinctions in patients with a common cold or similar acute respiratory illnesses can be made, using readily available information. There are no limits on the ethnicity, nationality, type of CC, or sex of the subjects; patients in different age ranges will be included in the study.

#### 2.1.3 Types of interventions and types of comparisons

Several methods of acup will be included in the review: fire needle therapy, fine needles, electric needle therapy, floating needle therapy, acupoint application, and acupoint injection. We will also include trials that evaluate acupuncture as a combination to other therapies. Multiple control interventions will also be included: no treatment, placebo, and other interventions (e.g., medicine and other physical interventions).

#### 2.1.4 Types of outcomes

Primary outcomes will include: the change in severity of overall symptoms of the common cold (e.g., absent, mild, moderate, severe); the change in duration of overall symptoms of the common cold (e.g., days to resolution).

Secondary outcomes will include: the change in severity of individual symptoms (e.g., nasal congestion, rhinorrhoea, sneezing); the change in duration of individual symptoms (e.g., nasal congestion, rhinorrhoea, sneezing); side effects from using acupuncture.

### 2.2 Search methods for identification of studies

#### 2.2.1 Electronic searches

Relevant databases include: Medline, Cochrane Library, Web of Science, EBASE, Springer, WHO International Clinical Trials Registry Platform (ICTRP), China National Knowledge Infrastructure (CNKI), Wanfang, Chinese Biomedical Literature Database (CBM), Chinese Scientific Journal Database (VIP). All RCTs published in electronic databases from inception to January 1, 2019 with language restricted in Chinses and English will be included in this review study. The Medline search strategy is listed in Table 1, which includes all search terms, and other searches will be conducted based on these results.

**Table 1:**
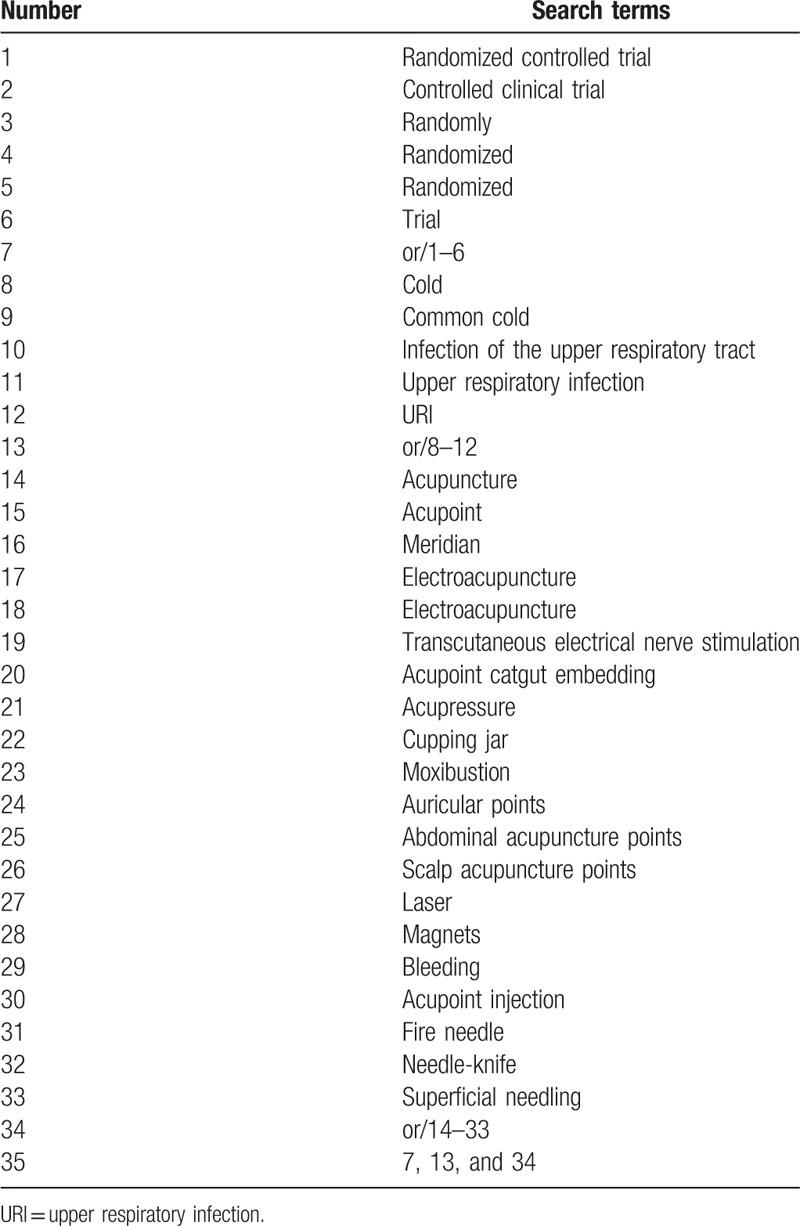
Medline search strategy.

### 2.3 Data collection and analysis

#### 2.3.1 Selection of studies

**Figure 1:**
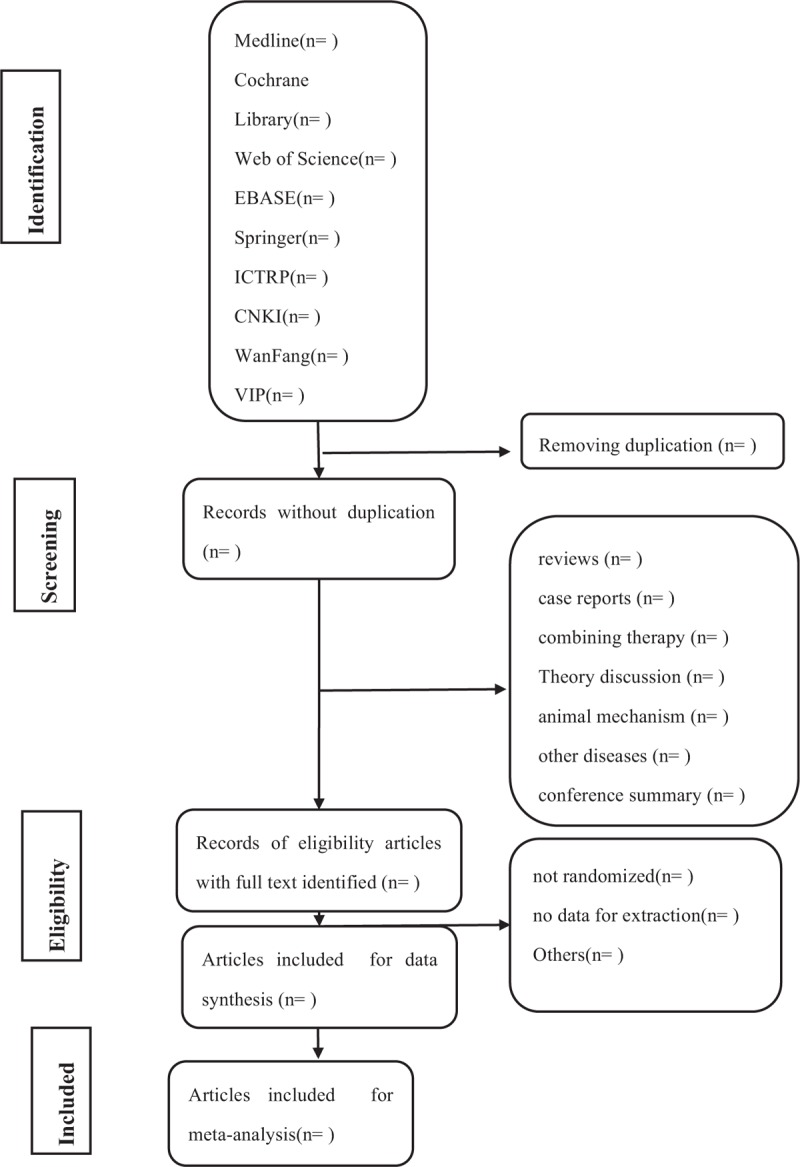
Flow diagram of studies identified.

Depending on the type of literature research, intervention measures, and object of study, we will rule out unrelated literatures in early screening. The study of screening flow diagram is summarized as Fig. 1.

#### 2.3.2 Assessment and quality of included studies

Investigators will evaluate quality of articles and assess the risk of bias based on the domains and criteria of the Cochrane Collaboration tool (RevMan V.5.3; Copenhagen, The Nordic Cochrane Centre, The Cochrane Collaboration, 2014).^[13]^ Two RCTs compared the effectiveness of acupuncture with a control. In addition, controversial problems will be resolved by consulting experts. Study quality will assessed independently by 2 investigators (YC and BG) including what randomized method has been used, whether the results of the data are complete and whether grouping scheme is hidden or not.

#### 2.3.3 Data extraction

Data will be extracted independently by 2 investigators (YJ and NX) using a data extraction sheet. The datum of each selected trial will be collected and recorded as a electronic form which includes that basic information of studies (numbering, which combines the first author's last name with year published, correspondence of author), sample size and grouping method; details of intervention methods include treatment time, selection of therapy, acupoints, data type of treatment efficacy, treatment cycles, ascertainment of outcome, side effects, and follow-up.

#### 2.3.4 Measures of treatment effect

A risk ratio (RR) with 95% confidence intervals (CIs) is used to estimate the overall outcome of treatment with dichotomous data. A standard mean difference (MD) or standard MD (SMD) with 95% CI will be presented for continuous outcomes. Other binary data will be altered into the RR form. This work will be performed by 2 independent persons to reduce inaccuracy in the extracted rates.

#### 2.3.5 Dealing with missing data

In view of the lack of data that needs to be extracted in the included document, if there is a statistical missing data, we will attempt to contact the authors by phone or email. Articles in which data are not given or the given information could not be computed will be excluded.

#### 2.3.6 Assessment of heterogeneity

To assess heterogeneity among the studies, we will use chi-squared test and *P* values, and the heterogeneity will be evaluated by *I*^2^ statistic. An interpretation of *I*^2^is as follows: *I*^*2*^ ≥50% will be considered a measure of severe heterogeneity, while *I*^*2*^ <50% will be presented as no heterogeneity. So the conclusion will be made with discretion. If the clinical and methodological heterogeneity are not presented, the stochastic effect model will be assessed by merger analysis. If the heterogeneity is oversized, descriptive analysis will be performed.

#### 2.3.7 Assessment of reporting bias

Funnel plots and statistic test will be used to detect reporting bias on overall estimate.

#### 2.3.8 Data synthesis

Based on the sample size of the clinical research including the research designing of measurement methods, intervention methods, and the length of treatment, we will perform the systematic review of the literature with meta-analysis. When multiple homogeneity studies are included, meta-analyses will be performed with Review Manager 5.3.5. When *I*^2^ < 50%, the fixed effect model will be selected and the random-effect model will be selected *I*^2^ ≥50%. Otherwise, we will exclude study from meta-analysis.

#### 2.3.9 Other analysis

If the heterogeneity is caused by clinical trials above-mentioned, subgroup analysis will be conducted, and detailed subgroup will be classified. If we identify substantial heterogeneity according to the outcomes of data synthesis, the following subgroup analyses plan to carry out.

(1) Different types of acupuncture therapies.
(2) Symptoms of CC (headache, fever, nasal obstruction, cough, and general pain with anhidrosis, etc.).

Sensitivity analysis: By changing the genre of research (incorporating or excluding a particular study), reanalysis of simulated missing data we will observe fluctuation of termination.

## 3 Discussion

CC comprises symptoms of headache, sneezing, soar throat, fever, nasal congestion, cough, and general pain with anhidrosis.^[14]^

Having the effect of promoting metabolism circulation, balancing, improving immunity, dredging meridian, and harmonizing qi and blood,^[15]^ traditional Chinese acupuncture shown to be effective and safe with strong operability, low cost, and environmental friendly, especially suitable for children with fever. Acupuncture is based on the theory of TCM guided by the theory of meridians. A present review suggests that a significantly positive benefit is obtained from the administration of acupuncture therapy.^[16]^ The use of acupuncture for symptoms of the common cold symptoms should be considered, although further evidence from placebo controlled RCTs is required.

According to Cochrane method, this research is based on the existing clinical RCT evidence analysis at home and abroad, retrieving and screening the main electronic literature database with evidence of evidence-based medicine, to better guide clinical practice.
